# Small Changes Huge Impact: The Role of Protein Posttranslational Modifications in Cellular Homeostasis and Disease

**DOI:** 10.4061/2011/207691

**Published:** 2011-07-21

**Authors:** Tejaswita M. Karve, Amrita K. Cheema

**Affiliations:** ^1^Department of Biochemistry, Cellular & Molecular Biology, Lombardi Comprehensive Cancer Center, Georgetown University School of Medicine, 3900 Reservoir Road, NW, Washington DC 20057, USA; ^2^Department of Oncology, Lombardi Comprehensive Cancer Center, Georgetown University School of Medicine, Washington, DC 20057, USA

## Abstract

Posttranslational modifications (PTMs) modulate protein function in most eukaryotes and have a ubiquitous role in diverse range of cellular functions. Identification, characterization, and mapping of these modifications to specific amino acid residues on proteins are critical towards understanding their functional significance in a biological context. The interpretation of proteome data obtained from the high-throughput methods cannot be deciphered unambiguously without a priori knowledge of protein modifications. An in-depth understanding of protein PTMs is important not only for gaining a perception of a wide array of cellular functions but also towards developing drug therapies for many life-threatening diseases like cancer and neurodegenerative disorders. Many of the protein modifications like ubiquitination play a decisive role in various drug response(s) and eventually in disease prognosis. Thus, many commonly observed PTMs are routinely tracked as disease markers while many others are used as molecular targets for developing target-specific therapies. In this paper, we summarize some of the major, well-studied protein alterations and highlight their importance in various chronic diseases and normal development. In addition, other promising minor modifications such as SUMOylation, observed to impact cellular dynamics as well as disease pathology, are mentioned briefly.

## 1. Introduction

With current advances in the fields of systems biology and proteomics, the interest in deciphering protein modifications and their impact on the cellular microenvironment and disease pathophysiology is greatly enhanced. Proteins are large macromolecules comprised of a specific sequence of amino acids. Although protein folding and refolding play a critical role in protein function, the modification of amino acids and their side chains contributes significantly to the structural and functional diversity of the proteins. These modifications impart complexity to the eukaryotic proteomes that is several orders of magnitude greater than the coding capacity of the genome. The common modifications include phosphorylation, acetylation, glycosylation, ubiquitination, acetylation, and hydroxylation. Posttranslational modifications (PTMs) of proteins influence the enzyme activity, protein turnover and localization, protein-protein interactions, modulation for various signaling cascades, DNA repair, and cell division. 

Given the pivotal role of PTMs in the regulation of cellular environment, there is a constant effort to develop novel, highly sensitive, and sophisticated PTM identification techniques. Some of these techniques are targeted towards identifying specific PTMs like the modification of the histone tails recognized by specially designed probes while other techniques are more robust like surface-enhanced Raman spectroscopy and mass spectrometry [1–3]. A novel technology called “multidimensional protein identification technology or MudPIT,” which is a combinatorial method chromatography in conjunction with mass spectrometry, has been efficient in discovering global PTM [4]. Traditional biochemical methods like Western blotting and SDS-PAGE are widely used to confirm the high-throughput results obtained from the spectroscopic methods as well as for understanding the biological significance of PTMs *in vivo*. In addition, there have been successful attempts in developing *in silico* algorithms that can reliably predict various PTMs in a given protein sample. Other artificial PTMs like biotinylation, which attach a prosthetic group to a protein, are frequently used to understand protein-protein interaction(s) that results from the changes in three-dimensional structure of protein. Although more than 150 protein modifications have been reported, a detailed assessment of each modification is beyond the scope of this paper. We have focused on major modifications, which have received significant attention by the research community in the past few decades. 

## 2. Acetylation

One of the most common protein modifications is the acetylation of lysine residue. The acetylation of proteins is mainly a cotranslational and posttranslational process. The histone acetylation as well as deacetylation is of particular interest due to its role in gene regulation [5, 6]. This occurs on the lysine residue of histone proteins at the N-terminal tail of lysine and is facilitated by the enzymes, histone acetylases (HATs), or histone deacetylases (HDACs). A single lysine alteration on histones significantly impacts the cellular homeostasis since the acetylation status of histones regulates various transcription factors, molecular chaperones, and cellular metabolism [7, 8]. In addition, regulation of histone acetylation by the HATs and HDACs has a well-established link to aging and various neurological and cardiovascular diseases [9–12]. Finally, at least one HDAC family, MYST proteins, is shown to participate in a diverse array of functions in health and disease to modulate the fate of stem cells and chromatin state [13]. 

In prokaryotes, the acetylation of glutamic acid and aspartic acid has also been observed. The conversion of glutamic acid to N-acetylglutamic acid is an important intermediate step for ornithine synthesis in bacteria [14]. 

Recent studies not only have linked the process of protein acetylation with a number of diseases but also have shown that amino acid acetylation significantly contributes to the overall pathophysiology of the diseases [9–12]. One such study has noted that the increased acetylation of the cytoskeletal proteins, especially microtubule proteins, in response to the reactive oxygen species (ROS) and thus suppression of SIRT2 aggravates the mitochondrial dysfunction in the CPEO (chronic progressive external ophthalmoplegia) syndrome patients [15]. Conversely, the study showed that the lysine hyperacetylation of the OGG1 enzyme, an important DNA repair enzyme, in response to the ROS, is a required step for the activation of the DNA repair system [16]. Similarly, another member of the deacetylases family, SIRT1, has been shown to be downregulated in oxidative stress-induced endothelial cells. However, pretreatment with a pharmacological agent like resveratrol was shown to attenuate the SIRT1 levels as well as eNOS acetylation. Thus, identification of the eNOS as a substrate for SIRT1 in the endothelial cells has been a pivotal step in understanding the pathology and mechanism of the cardiopulmonary diseases and vasculature [17]. 

Acetylation of proteins and carbohydrates has also been evaluated as a target for cancer therapy [9, 18]. Chammas et al. have reported the use of N-acetylation as well as O-acetylation of the surface tumor antigens as a tool for therapeutic development against different types of melanomas and leukemias [9].

A number of acetoproteins have been implicated in the cognitive disorders like dementia and Alzheimer's disease [10]. In the case of dementia, lysine acetylation of tau proteins results in “tau tangles” while in Alzheimer's disease lysine hyperacetylation of *β*-amyloid peptide results in impaired cognition [10]. Additionally, mouse models have demonstrated that alterations in histone acetylation pattern play a role in age-dependent memory impairment [11]. Thus, these studies indicate that targeting lysine acetylases and deacetylases might be promising avenue for developing novel therapies for neurodegenerative diseases. The widespread and dynamic nature of lysine acetylation and the nexus that exists between epigenesis-directed transcriptional regulation and metabolism has been comprehensively reviewed [19]. 

## 3. Carbonylation

Protein carbonylation can result from excessive oxidative stress in a biological system. It is an irreversible PTM which may lead to the formation of nonfunctional proteins, in turn leading to many diseases. Many disorders including autoimmune diseases and cancer are mediated by increased production of reactive oxygen as well as nitrogen species (ROS and RNS) in the cell. However, the role of ROS and RNS in protein oxidation is less understood. Protein carbonylation has received significant attention as an indicator of oxidative or genotoxic stress [20, 21]. Investigation of the Murphy Roth's Large (MRL) mouse model, used widely in tissue regeneration studies, suggested that common environmental contaminants like trichloroethene (TCE) increased ROS and RNS leading to the production of high amounts of nitrotyrosine and protein carbonyl(s), a classic hallmark response to high oxidative stress [22]. These byproducts are seen to induce a number of autoimmune diseases like systemic sclerosis and fasciitis. Elevated levels of protein carbonyls and nitrotyrosine have also been observed in other instances with high oxidative stress, mitochondrial disorders, indicative of hypocitrullinemia and loss of glutathione (iGSH) [23]. Thus, carbonylation of amino acids like proline, arginine, lysine, threonine, glutamate, and aspartate (see Table 1) is irreversible and results in a non-functional protein, which then participates as a mediator in a number of chronic diseases, especially the ones that are influenced by the status of oxidative stress in a cell [24]. A recent proteomics study has detailed the identification of carbonylation pattern as a fingerprint for oxidative stress [25]. The carbonylation pattern can thus be an informative tool for the identification of stress as well as a marker for therapy for many mitochondrial, neurological, and cardiovascular diseases. Another proteomics study conducted in mouse model for an early stage of alcoholic liver disease (ALD) identified biomarkers for early stage ALD wherein a carbonylated protein expression pattern was highlighted [26].

At least one study that focused on sepsis in mouse models concluded that N-acetylcysteine, an antioxidant, induces cellular antioxidant defense and prevents nitration of tyrosine residues and protein carbonylation. These results indicate the therapeutic potential of N-acetylcysteine for treating sepsis patients [27]. Protein carbonylation, particularly of UCH-L1 forming carbonyl-modified UCH-L1 and its interaction with other proteins like tubulin, was thought to be one of the causes of familial as well as sporadic Parkinson's disease (PD) [28]. The carbonyl-modified UCH-L1 is proposed to be an investigative tool to explore the underlying molecular mechanism of PD development [28]. This carbonyl modification may be of therapeutic value targeting the familial and/or sporadic PD [28]. Choi et al. demonstrated the irreversible carbonylation of protein to methionine sulfone as an indicator of oxidative stress damage in cases of the sporadic PD and Alzheimer's as well as other neurodegenerative diseases [29]. In recent years, proteomic tools and methods for the identification of sites of protein carbonylation have been widely developed [30]. 

## 4. Glycosylation and Glycation

Another well-studied cotranslational and posttranslational mechanism is the addition of a sugar moiety to proteins, lipids, or other organic molecules inside or outside of the cell. Glycosylation being an enzyme-directed reaction is site as well as substrate specific, tightly regulated and reversible. On the other hand, glycation is a random event that most often leads to the formation of defective or non-functional biomolecules. 

Glycans resulting from glycosylation are classified under one of five known classes: N-linked glycans, O-linked glycans, C-linked glycans, phosphoglycans, and glypiation (GPI-anchored). O-linked glycans have been shown to participate in the diverse cellular processes and development [31, 32]. Glycosylation, a covalent modification, plays a central role in protein localization, protein-protein interactions, structural stability of the cell, immune responses, and modulating of cell signaling [32, 33]. Thus, any dysfunctional glycans formed in the cell could lead to diseases including cancer, liver cirrhosis, diabetes, and exacerbated HIV infection [34, 35]. A novel glycosylation prediction tool developed by Szabá et al. utilizes currently available databases of the T-cell antigens and autoantigens glycosylation [36]. 

O-glycosylation, as well as phosphorylation, has been shown to have a beneficial effect in Alzheimer's disease by reducing the formation of neurofibrillary tangles in neurons [37]. Glycosylation of prion (PrP), a cell surface protein and a transmissible agent, is a determinant of the final disease outcome in the host [38]. Recent characterization of glycosylation sites on apolipoprotein E (apoE) revealed a novel glycosylation site in addition to the already known sites as well as at least 8 new complex glycans in secreted and cellular apoE [39]. The involvement of apoE in Alzheimer's disease, atherosclerosis, and immune responses is well documented, and this novel information can help gain insight towards understanding the mechanistic role of glycosylated apoE residues in these diseases [39]. Improper or incomplete glycosylation in the Fc receptor for immunoglobulin A has been shown to impact the IgA-mediated immune response which in turn affects many diseases including HIV, alcoholic liver cirrhosis, and other neuropathies [34, 40]. 

Other glycation products called (AGEs) have been implicated in cardiovascular diseases, cataract, and diabetes mellitus apart [41, 42]. The end products are commonly used as markers to evaluate the disease prognosis since inhibiting a subclass of the AGEs has been shown to benefit physiological conditions like diabetes and arthrosclerosis [43–46]. Finally, glycosylation is shown to be a contributing factor in cancer cell transformation via Src(s) as well as in regulating various signaling pathways like Wnt-*β* catenin pathway, thereby affecting the disease physiology and final outcome [47]. 

## 5. Hydroxylation

Hydroxylation is an important detoxification reaction in the cell and is mostly facilitated by the group of enzymes called hydroxylases. It is also one of the few reversible, post-translational modifications and hence has a prominent relevance to the cellular physiology. 

Proline hydroxylation-mediated modification of collagen has been studied extensively since it has significant implications on the structural physiology of the cell [48]. Some cancers or metabolic disorders like scurvy are linked to the lack of proline hydroxylation due to ascorbate deficiencies, an important component of the reaction [49]. The enzyme prolyl 4-hydroxylase that catalyzes the conversion of 4-hydroxyproline to collagen, is one of the most well-studied enzymes in this group [50, 51].

Proline hydroxylation is an important step in activating antioxidant defense against hypoxia via hypoxia inducible factor (HIF) [52]. Under normoxia, proline hydroxylation acts as a regulatory step for HIF1*α* and 2*α* to bind to the von Hippel-Lindau tumor suppressor (pVHL) E3 ligase complex, which targets both the factors for rapid degradation by ubiquitin-proteasome complex [52]. Under hypoxic conditions, however, the abrogation of proline hydroxylation as well as asparagine hydroxylation is necessary for the continual action of the HIF transcription factor. Thus, hydroxylation of asparagine and proline acts as a “hypoxic switch” for induction of HIF under the low oxygen conditions [53]. Further, proline hydroxylation of HIF similar to that under normoxic conditions was shown to be protumorigenic [54, 55]. The inhibition of normoxic HIF1*α* was suggested as a therapeutic alternative in such cases [55, 56]. A number of *in silico* prediction tools augment the understanding of the complexity of the process as well as designing novel therapies for diseases like cancer and cholestatic liver disease [57]. 

Other amino acids that undergo hydroxylation include phenylalanine, tyrosine, and tryptophan, all of which have aromatic side chains [58]. Several genetic diseases are linked to the lack of hydroxylation of aromatic amino acids like that of phenylketonuria (PKU) and hyperphenylalaninemia, due to a defect in phenylalanine hydroxylase, an enzyme that converts phenylalanine to tyrosine [59]. Tyrosine hydroxylase is used as a molecular target to treat hypertension [60–62]. This enzyme is also known to act as an autoantigen in autoimmune polyendocrine syndrome (APS) type I. On the other hand, tryptophan hydroxylation, catalyzed by tryptophan hydroxylase, is a critical regulatory step in the production of an important neurotransmitter, serotonin [63]. 

## 6. Methylation

Protein methylation has a tremendous impact in health and disease, spanning from embryonic to postnatal developmental stages in numerous physiological conditions such as cancer, lipofuscinosis, and occlusive disease [64–68]. The most commonly methylated amino acid residues are lysine and arginine with lysine methylation receiving special consideration due to its role in epigenetics and chromatin remodeling [69]. 

Histone methyltransferases (HMTs), also sometimes referred to as the histone lysine methyltransferases (HKMTs), specifically target lysine residues in histones, which regulate gene expression [69]. Histone methylation together with the histone acetylation also has been shown to control cellular RNA synthesis including activation and inhibition of specific RNAs types, metabolism, and degradation *in vivo* [69, 70]. 

**Figure 1:**
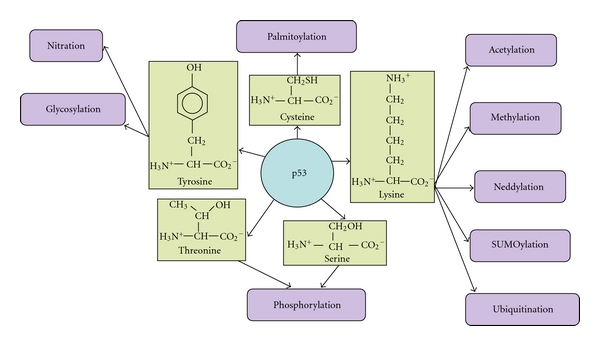
Post-translational modifications for p53, a tumor-suppressor protein, responsible for maintaining the genomic stability in a cell. The figure illustrates various posttranslational modifications that are frequently observed in p53 with varied functional implications in normal and/or diseased condition. The amino acid residues that most often undergo the respective modifications in a given context have been highlighted. For details of posttranslational alterations in p53 refer to [113–127].

In addition, there is a growing number of lysine methyltransferases that methylate nonhistone proteins on lysine residue that are being constantly identified [69]. These can methylate proteins like p53 (Figure 1), ER*α*, NF-*κ*B, and pCAF and other transcription factors that have been implicated in tumorigenesis and other metabolic disorders like inflammatory and immune responses. In addition to regulating gene expression, they regulate the protein stability by dominating the downstream effector responses that are responsible for the cell fate [69]. 

Postnatal developments like erythropoiesis, development of immune system, and other cell signaling cascades are regulated not only by chromatin methylation/demethylation but also by targeting specific amino acid residues for post-translational modification(s) in a given context [64, 65, 71].

The role of methyltransferases in various diseases has been widely noted. In the case of lysosomal storage diseases, methylation of a specific lysine of the mitochondrial ATP synthase plays central role in the accumulation and storage of this protein in the form of aggregates in the lysosomal bodies [68]. Levels of homocysteine, a methylation product of methionine, plays a major role in cardiovascular diseases as well as neurological disorders like Parkinson's disease [66, 72]. These diseases are exacerbated by the excessive accumulation of homocysteine due to the lack of removal or inefficient metabolism of this compound [72]. However, optimal amount of homocysteine is necessary for the normal functioning of the body, and its metabolism is extremely sensitive to internal vitamin B levels. [72]. Therapeutic approaches targeting plasma homocysteine are being proposed to counter the harmful effects of elevated homocysteine in patients [72]. Inaccurate methylation of oncoproteins in various cancers is commonly observed in conjunction with the upregulation of many lysine demethylases [67]. 

## 7. Nitration

Protein nitration and carbonylation are the by-products of the protein oxidation reactions. Nitration is a reversible and a stable post-translational modification that is initiated when amino acids are exposed to nitrating agents or oxidative stress [59]. Formation of protein carbonyls and nitroderivatives is classic hallmark of exposure to genotoxic stress [73]. Nitroproteins are thought to be involved in a plethora of diseases. Nitrotyrosine, a chief nitration product, is associated with many neurodegenerative diseases, lung diseases, inflammation, cardiac diseases, and cancer [74]. In addition, nitrotyrosine has been implicated in the regulation of various cell signaling pathways thus activating or inhibiting certain cellular transduction signals depending on the context of cellular physiology at any given time [75, 76].

A recent study has highlighted that the conversion of a tyrosine 253 of HDAC2 to nitrotyrosine not only abrogates its activity in the cell but also targets it for rapid proteasomal degradation and ultimately affects the gene regulation in the cell. This study is particularly important for novel cancer therapies that explore the option of HDAC inhibitors [77]. Furthermore, this study proposes a mechanism explaining the underlying effect of nitrosative stress in the context of a neoplastic transformation. It should be noted that certain flavonoids have been shown to inhibit protein nitration as well as induce cellular antioxidant defense response [78, 79]. Such phytochemicals seem to have a potential therapeutic value, especially in cases of cancer and ischemic retinopathy. At least one mechanistic study has provided strong evidence in recommending dietary supplements like epicatechin and N-acetylcysteine (NAC), both of which inhibit tyrosine nitration, to alleviate diabetic retinopathy and ischemic retinopathy that result from excessive nitrosative stress [79]. 

An *in vitro* mass spectrometric analysis of Lewy bodies in Parkinson's disease was able to capture the classical hallmark of the disease, an increase in 3-nitrotyrosine (3-NT) modification for various proteins [80]. Such elegant studies not only further our understanding of protein nitrosylation but also shed light on the intrinsic complexity of this modification that can be useful in designing targeted therapy for such a debilitating neurological malady [81]. 

Additionally, 3-NT and nitrated A2E proteins have recently been proposed as biomarkers for age-related macular degeneration (AMD) since their accumulation increases with age and specifically with increased nitrative stress [82]. 3-NT is routinely used as a biomarker for protein damage that is induced by oxidative inflammation [83]. Continual overproduction of nitric oxide or NO is a classical marker of the cellular inflammation, and this overproduced NO not only can damage the organelles but is also a contributing factor for cell death mainly by mediating apoptosis [84]. Nitrative controlled apoptosis in hepatic stellate cells (HSCs) is crucial because protein nitration plays a significant role in liver fibrosis prognosis [84].

Peroxynitrite, another harmful nitration product formed from NO and superoxide ion, along with nitrotyrosine was shown to contribute to amyloid *β*-peptide-induced toxicity and tau protein neurofibrillary tangles in Alzheimer's patients [85]. Peroxynitrite and cellular nitroproteins are also linked with inflammation of human colonic epithelium, a symptom of irritable bowel syndrome (IBS) [86]. 

## 8. Palmitoylation

Palmitoylation is a unique reversible cysteine thioaylaction modification that consists of covalent attachment of the fatty acid, mainly palmitic acid, to the protein molecule [87, 88]. This modification is unique, partly because of its reversible nature as compared to the other lipid-protein modifications like prenylation/isoprenylation and myristoylation, both of which are irreversible and co-translational reactions. However, these reactions tend to make proteins more hydrophobic as they add lipid molecule(s) to the protein structure. These modifications are also involved in cell trafficking, membrane stimuli, and protein-protein interactions [87, 88]. Palmitoylation was shown to play a regulator role in the G-protein linked cell signaling pathways via modification of the regulators of G protein signaling (RGS) proteins [89]. Palmitoylation of a specific cysteine residue in the RGS protein not only regulates the activity of the protein but also is an important mediator for localization and targeting of certain G-proteins and for modulating G-protein signaling [89]. However, in the case of thyrotropin-releasing hormone (TRH) receptor type 1 (TRH-R1), palmitoylation is not required specifically for G-protein signaling but for maintaining the inactive form of the receptor, since the unpalmitolyated form is constitutively active and leads to oversecretion of thyrotropin and prolactin [90]. Apart from the G-protein trafficking, palmitoylation is closely involved in the neural inflammatory response including demyelinating diseases as well as T-cell autoimmune responses [91]. Hence, a novel approach using the stability of palmitoylated proteins to present as antigens to the MHC-class II immune response is being proposed in order to benefit the patients with autoimmune diseases including multiple sclerosis [91]. 

Reduced or dysfunctional palmitoylation has been linked to many diseases and disorders [92–96]. In diabetic vascular disease, efficient palmitoylation of the endothelial nitric oxide synthase (eNOS) is a required step for efficient bioavailability of eNOS so it can be targeted to the plasma membrane [93]. Lack of eNOS palmitoylation, as observed in insulin-deficient or insulin-resistant patients, leads to the chronic inflammatory response in these patients [93]. Inefficient palmitoylation of the *γ*-secretase, a component of the *β*-amyloid aggregate, in Alzheimer's disease adversely affects the proper trafficking and functional potential of the neurons [92]. Again, lack of the distinct cysteine palmitoylation of the Huntington protein (HTT), a player in Huntington's disease, increases neural toxicity and enhances rapid formation of inclusion bodies, further deteriorating the patient prognosis [94].

In some of the disorders, protein palmitoylation can worsen the disease outcome. Cysteine 172 residue of Hepatitis virus C core protein (that forms the viral nucleocapsid) undergoes an essential step of palmitoylation, in order to efficiently multiply the virion particles and thus sustain an active infection in the host cells [95]. Palmitoylated oncogenic NRAS is a proposed target for developing therapies against NRAS-associated malignancies like acute myeloid leukemia (AML) as well as other types of NRAS-amplified leukemias [96]. With such a contradictory role for the palmitoylation, researchers have developed novel probes that can be utilized for the fluorescence microscopy and mass spectrometry analysis of protein palmitoylation [97]. 

## 9. Phosphorylation

Phosphorylation, addition of a phosphate group to an amino acid, is one of the central reversible, post-translational modifications that regulate cellular metabolism, protein-protein interaction, enzyme reactions, and protein degradation for a myriad of proteins, which results in intracellular signaling cascades [98, 99]. This reaction is mediated by a number of protein kinases (PKs) in the cell. Conversely, dephosphorylation or removal of a phosphate group is an enzymatic reaction catalyzed by various phosphatases (PPs) [98]. The ERK1/ERK2-MAPK signaling (mitogen-activated protein kinase), a central cell proliferation pathway which intercepts with the receptor tyrosine kinases (RTKs) pathway, and cell cycle progression proteins like cyclin-dependent kinases (CDKs) are some of the networks that are affected by the phosphorylation/dephosphorylation status of proteins [98]. Thus, a proper balance of action between the PKs and PPs is a key to maintain cellular homeostasis. Autophagy, a cell death mechanism, is also phosphorylation-dependent [100]. At least, one report has shown that the autophosphorylation event of the Atg1 protein is a “regulatory switch” that determines the initiation of the process [100]. 

Serine/threonine (Ser/Thr) and tyrosine are the most commonly observed phosphorylated amino acid residues and have been frequently implicated in progression of cancers [101–103]. For example, okadaic acid present in shell fish poisoning rapidly stimulates Ser/Thr phosphorylation in an intact cell while simultaneously inhibiting many phosphatases thus inducing phosphorylation-mediated signaling cascades which promote uncontrolled cell proliferation [102, 104]. Dysregulated phosphorylation has been implicated in neurological diseases like Parkinson's and dementia that harbor the accumulation of the Lewy bodies [104]. Ser-129 phosphorylation of *α*-synuclein protein is responsible for the build-up of proteolytic Lewy aggregates [104]. In case of lung cancer, at least one distinct threonine (T163) phosphorylation event on the protein Mcl-1, induced by nicotine (an active ingredient of tobacco), is responsible for chemoresistance in these tumors [105]. Thus, a single phosphorylation event in this case is responsible for cell survival due to blocking of the antiapoptotic function of the protein Mcl-1 and promoting tumorigenesis. The role of protein phosphorylation/dephosphorylation in cancers and their huge impact in disease pathophysiology has been extensively reviewed multiple times over the years, with a steady stream of new discoveries of protein phosphorylation and their effects in disease pathology [106–109]. The phosphorylation of eNOS, on the other hand, is a key to its own regulation that is central in many inflammatory and autoimmune diseases [110]. The regulation of the NF-*κ*B cascade, which controls chemokine and cytokine responses and inflammation, is activated in response to the stimuli via phosphorylation. This abnormal phosphorylation of the NF-*κ*B cascade is a classical hallmark of cancers and chronic immune disorders [111]. Thus, the molecular targeting of specific kinases and phosphatases is seen as a promising strategy in treating cancer as well as other inflammatory diseases [111, 112]. 

The effects of the process of protein phosphorylation on cell physiology are enormous. As such, it is beyond the scope of this paper to cover every aspect of this ubiquitous modification. However, we have made an effort to highlight few important features of phosphorylation itself as well as combined effects with other PTMs. 

## 10. Sulfation

N-sulfation or O-sulfation, facilitated by the addition of a sulfate group by oxygen or nitrogen, respectively, is another post-translational protein modification, commonly observed for membrane as well as secreted proteins [128–130]. Sulfated proteins have been observed to play a role in protein-protein interactions, G-protein receptor signaling, chemokine signaling, and immune responses [131, 132]. However, their precise role in cellular regulation still remains somewhat enigmatic [129]. Gao et al. showed that tyrosine sulfation was involved in cellular calcium transportation and mediating association between the chemokine receptor (CXCR3) and IFN *γ*-inducible protein-10 (IP-10) [133].

As mentioned above, tyrosine sulfation is a key player in many diseases including autoimmune response, HIV infection, lung diseases, multiple sclerosis, and cellular enzyme regulation [131, 134–137]. Heavy sulfation of high-molecular weight glycoconjugates (HMGs) produced by cystic fibrosis (CF) respiratory epithelia was shown to adversely affect the association between HMG and airway secretions and possibly create a breeding ground for harmful bacteria like *P. aeruginosa* and *S. aureus* in the CF airways and thus contributing to the pathogenesis of the disease [136]. Other lung diseases like chronic obstructive pulmonary disease (COPD) and asthma are equally aggravated due to induction of chemokine signaling by the tyrosine sulfation and thus affecting the downstream molecular players along with the leukocyte trafficking and airway inflammation [135, 138].

Tyrosine sulfation still remains one of the major posttranslational modifications that is involved in multiple disorders [131, 135, 136, 138]. Thus it is also being proposed as a molecular target for developing a prophylaxis against HIV1 infection as it greatly diversifies the antigen availability and presentation beyond the standard 20 amino acids [134]. Given its role and potential as a target for the drug development, there are few sulfation site(s) prediction tools, like that of random forest algorithm, being tested [139]. Techniques like mass spectrometry that are highly sensitive and specific for “sulfoproteome” analysis due to the presence of the sulfoester bond in the sulfated amino acids are frequently used [129, 140]. 

## 11. Ubiquitination

Ubiquitination is a highly dynamic, coordinated, and enzymatically catalyzed post-translational modification that targets proteins for degradation and recycling [141]. Proteins that are targeted for degradation are tagged by a covalent attachment of a small regulatory protein, ubiquitin (Ub) [141, 142]. This process is called ubiquitination which is a multistep enzymatic process [142]. It consists of three main enzyme classes that act in a specific order: Ub activating enzymes (E1), Ub conjugating enzymes (E2), and Ub ligases (E3) [143]. Proteins targeted for degradation can be mono-Ub or poly-Ub which is dependent on the type and localization of the substrate. Some of the notable multi-subunit E3(s) are anaphase-promoting complex (APC) and the SCF complex (Skp1-Cullin-F-box protein complex) that generally destine the target protein for the proteasomal degradation. 

Another group of proteins called ubiquitin-like proteins (ULPs), which also follow the traditional path of sequential E1-E2-E3 processing for undergoing ubiquitination modification, need a mention [144]. They modify cellular targets in a pathway that is parallel to Ub but they maintains its distinctiveness [144]. Three of these ULPs that have received a lot of attention are NEDD8, Sentrin/SUMO, and Apg12 [144]. Ubiquitination is critical in almost every cellular process as well as a major player in almost any disease or disorder. Ubiquitination has a role in modulating diverse cellular functions like cell proliferation and differentiation, autophagy, apoptosis, immune response, DNA repair, neural degeneration, myogenesis, and stress response [145–147]. It also affects the outcome of many life-threatening diseases like cancer, neurodegenerative disorders, HIV infection, Herpes, and liver diseases [148–151]. A bi-functional ubiquitin editing protein called A20 was shown to regulate NF-*κ*B signaling, affecting gene transcription, cell proliferation, and inflammatory responses [147]. It is also linked with the inhibiting Beclin-1 ubiquitination, an autophagy inducer protein, thus limiting the autophagic response [145]. A transcription factor that regulates the cellular antioxidant defense, NFE2L2, is stabilized and thus protected from the Ub-proteasomal degradation via six conserved cysteine residues on the N-terminal of the NFE2L2 [128]. NFE2L2-mediated gene regulation has been proposed as a therapeutic alternative not only in cancer but also for neurodegenerative diseases that show high oxidative stress like Batten's and Parkinson's diseases [152, 153]. Even though, these studies propose usage of ubiquitination inhibitors that are effective in cancer and neurological diseases as they stabilize tumor suppressor proteins and antioxidant defense mediators, the process of proteasomal degradation can be equally helpful in alleviating these diseases [154, 155]. One of the examples where ubiquitination might be beneficial is the proteasomal degradation of nuclear as well as oncogenic I*κ*B protein, a player in the NF-*κ*B signaling [147]. However, there are at least a few drugs, for example, Bortezomib, an FDA approved cancer treatment drug, that are inhibitors of ubiquitination and commonly used as a part of the treatment regimen for the cancer and other neural diseases [154]. 

## 12. Conclusion

Despite considerable efforts to understand the relevance of posttranslational modifications in the cellular context, we are still in the process of unraveling the complexity of these modifications and their tremendous impact. Sophisticated technological advances like high resolution mass spectrometry and reliable *in silico* tools are now increasingly available for identification and characterization of these site-specific protein alterations. One such novel application is the role of glycosylation resulting in the formation of disorderly proteins or intrinsically unstructured proteins (IUPs). Disorderly proteins are newly discovered proteins that are heavily modified by the post translational mechanisms resulting in non-functional or dysfunctional protein molecules. These proteins have been shown to play an essential role in gene transcription, protein expression, enzyme activities, cell signaling cascades, and so forth. Due to their role in disease pathology and cellular homeostasis, these protein molecules are actively sought after molecular targets for developing drugs for cancer treatment as well as other chronic diseases. Other well-known protein modifications like phosphorylation are key players in expanding the avenue of translational medicine for heterogeneous diseases like cancer. 

With the constant addition of new post-translational modifications, verification of newly identified proteins changes by traditional methods and correlating the biological significance is a challenging task. We are just beginning to grasp the enormity of the field and its effect on the normal development and disease pathophysiology. 

Continued search and evaluation of various functional modifications of proteins and understanding their interaction in various biological pathways have important implications in the successful development of novel prognostic markers as well as therapeutic targets for cancer, severe neurodegenerative diseases, and other debilitating genetic disorders. 

## Figures and Tables

**Table 1:** Comparative analysis shown for 20 amino acids with possible functional modification(s) observed for each amino acid residue. For more information, refer to the text and citations therein.

Amino acids	Acetylation	Carbonylation	Glycosylation and glycation	Hydroxylation	Methylation	Nitration	Palmitoylation	Phosphorylation	Sulfation	Ubiquitination
Alanine	*√*									
Isoleucine	*√*									
Leucine	*√*									
Valine	*√*									
Phenylalanine				*√*						
Tryptophan			*√*	*√*		*√*				
Tyrosine			*√*	*√*		*√*		*√*	*√*	
Asparagine			*√*	*√*						
Cysteine		*√*					*√*			
Glutamine					*√*					
Methionine					*√*					
Serine	*√*		*√*		*√*			*√*		
Threonine	*√*	*√*	*√*					*√*		
Aspartic acid	*√*	*√*		*√*						
Glutamic acid	*√*	*√*								
Arginine	*√*	*√*	*√*		*√*					
Histidine	*√*	*√*			*√*					
Lysine	*√*	*√*		*√*	*√*					*√*
Glycine	*√*									
Proline	*√*	*√*		*√*						
